# Compliance with intermittent preventive malaria treatment in pregnancy at a tertiary hospital in Sierra Leone

**DOI:** 10.5588/pha.25.0049

**Published:** 2026-05-18

**Authors:** W.K. Lahai, A.O. Vandy, K.C. Prajitha, B.D. Fofanah, E.M. Kamau, H. Owusu, J.S. Squire, H.A. Barrie, I.F. Kamara, A.M. Falama, N. Sesay, A.R.Y. Kamara, F. Kanu, M.S. Kanu, M.M. kamara, M.A. Sesay, J.A. Koroma, F. Moses, S. Lakoh, S. Kenneh, L.F. Grant

**Affiliations:** 1 National Malaria Control Programme, Ministry of Health, Freetown, Sierra Leone;; 2 World Health Organization Country Office, Freetown, Sierra Leone;; 3 Centre for Operational Research, International Union Against Tuberculosis and Lung Disease, Paris, France;; 4 UNICEF, UNDP, World Bank, WHO Special Programme for Research and Training in Tropical Diseases (TDR), Geneva, Switzerland;; 5 Korlebu Teaching Hospital, Accra, Ghana;; 6 National Public Health Agency, Freetown, Sierra Leone;; 7 Ministry of Health, Freetown, Sierra Leone;; 8 College of Medicine and Allied Health Sciences, University of Sierra Leone, Freetown, Sierra Leone.

**Keywords:** antenatal care, malaria in pregnancy, preventative treatment, implementation

## Abstract

**OBJECTIVES:**

To describe receipt of intermittent preventive treatment for malaria in pregnancy (IPTp) doses, assess compliance with the recommended minimum of three IPTp doses, and identify factors associated with non-compliance among antenatal care attendees. This was a non-concurrent cohort study using routinely collected hospital data in 2024 at the Princess Christian Maternity Hospital, a university-affiliated tertiary teaching hospital in Freetown, Sierra Leone.

**RESULTS:**

Among the 733 pregnant antenatal care attendees, 682 (93%) registered after the first trimester, and only 30 (4%) had ≥4 antenatal contacts. Of the 733, 406 (55%) received first dose and only 62 (8%) received three doses. The maximum number of doses received was six by a single woman. In adjusted analysis, women with less than four antenatal contacts had three times higher risk of non-compliance (adjusted relative risk: 2.7, 95% confidence interval: 1.6–4.5) compared with those with ≥4 contacts. Receipt of IPTp doses and compliance with the recommended minimum of three doses were low and were associated with fewer antenatal care contacts.

**CONCLUSION:**

Our findings underscore the need to understand the reasons for the gaps in treatment and to address them systematically. Strategies to increase the number of antenatal care contacts may improve compliance with the recommended minimum three doses.

Malaria in pregnancy can lead to maternal anaemia, low birth weight, stillbirth, spontaneous abortion, preterm delivery, and maternal death.^1^ According to the World Malaria Report 2024,^2^ pregnant women are at an increased risk of malaria infection in sub-Saharan Africa (SSA). Malaria in pregnancy is estimated to account for 20% of stillbirths and 11% of all newborn deaths in SSA, further worsening the region’s high neonatal mortality rates.^1^ Among the estimated 35.4 million pregnancies in 33 endemic countries of the WHO African Region, 12.7 million (36%) were exposed to malaria.^1^ In areas with moderate to high transmission of *Plasmodium falciparum*, the WHO recommends administration of at least three doses of intermittent preventive therapy in pregnancy (IPTp) using sulphadoxine 500 mg–pyrimethamine 25 mg (SP), until delivery.^3^ The first dose of IPTp-SP is recommended to be initiated as early as possible in the second trimester during routine antenatal care (ANC) contact, and continued at monthly interval until delivery to protect the mother and child from malaria.^3^

Sierra Leone is a malaria-endemic country with stable, perennial transmission, and *Plasmodium falciparum* accounts for approximately 95% of infections. Considering the increased risk of malaria infection among pregnant women in the country, IPTp was introduced in 2017 and is administered as directly observed therapy during ANC contacts at health facilities or through outreach.^4^ Compliance with the WHO recommended minimum of three doses of IPTp remains a challenge in the African region due to poor adherence, inconsistent access to health care services, limited awareness, and cultural barriers.^1^ In a recent study conducted in 33 endemic countries of the WHO African Region, the proportion of pregnant women receiving at least one dose (IPTp1) and three doses (IPTp3) of IPTp was 64% and 42%, respectively.^5^ In Sierra Leone, national data indicate a progressive decline in receipt across successive IPTp doses.^6^ The yearly target of 80% IPTp3 coverage set in the National Malaria Elimination Strategic Plan (2021–2025) is yet to be achieved.^6^ This gap is particularly evident in the Western Area Urban District, where only 54% of pregnant women received three or more doses.

Limited studies have examined IPTp dose receipt and compliance with the recommended minimum of three doses in Sierra Leone. The lack of comprehensive data on patterns, coupled with a limited understanding of factors associated with non-compliance, impedes efforts to optimise preventive strategies for malaria in pregnancy. Therefore, we conducted this study at the Princess Christian Maternity Hospital (PCMH), a referral maternity facility in the Western Area Urban District, to describe receipt of IPTp doses during ANC contacts in 2024 and to assess compliance with the recommended minimum of three IPTp doses. Addressing these gaps may help inform strategies to strengthen delivery of IPTp within routine antenatal services and improve maternal and neonatal health outcomes. The specific objectives of the study were 1) to determine the number and proportion of ANC attendees who received one, two, and three or more IPTp doses, including the period of initiation of IPTp, and 2) to identify factors associated with non-compliance with the recommended minimum of three IPTp doses.

## METHODS

This was a non-concurrent cohort study using routinely collected hospital data from the 1 January to 31 December 2024.

### Setting

Sierra Leone is located on the West Coast of Africa with a projected population of 8.8 million.^7^ The public health care system comprises 18 district hospitals and 1,463 peripheral health units (PHUs). The PHUs provide primary health care, whereas district hospitals provide secondary or tertiary health care. PCMH is a national referral and university-affiliated tertiary teaching hospital in Freetown, with a bed capacity of 136 and approximately 450 staff. The hospital serves a catchment population of more than 1.5 million people. Services offered at PCMH under Obstetrics and Gynecology include ANC, reproductive health and family planning, caesarean sections, pharmacy, blood bank, ambulance services, human immunodeficiency virus counselling and testing, and cervical cancer screening.

### National Malaria Control Programme (NMCP)

In 1994, the Ministry of Health, with technical support from WHO, established NMCP within the Directorate of Disease Prevention and Control to plan, coordinate, supervise, monitor, and evaluate all malaria prevention and control interventions.^8^ IPTp-SP is provided free of charge to all eligible pregnant women attending ANC at public health facilities nationwide.

### Antenatal registration and supply of IPTp-SP doses at PCMH

A pregnant woman attending the ANC unit is registered by a nurse midwife. During the process of registration, the nurse midwife records general information like name, age, address, and education in general ANC registers, and key obstetric information including gravida, parity, gestational age at registration, and maternal weight is recorded in the mother and neonate register. IPTp-SP is administered under directly observed therapy to pregnant women in their second and third trimesters, in accordance with national and WHO guidelines. Doses administered are recorded in the mother and neonate register.

### Study population

All pregnant women who registered and attended at least one ANC contact at PCMH between January and December 2024 were included.

### Data source and variables

Data were collected from the ANC registers and mother and neonate register at PCMH. Variables included were socio-demographic and obstetric characteristics such as age, marital status, religion, occupation, parity, gravida, gestational age at first ANC contact, and total number of ANC contacts. Total number of ANC contacts was categorised as <4 versus ≥4 contacts for regression analysis.

Receipt of IPTp doses was defined as pregnant women receiving one or more doses during ANC contacts and categorised as receipt of one dose (IPTp1), two doses (IPTp2), and three or more doses (IPTp3). Compliance was defined as receipt of three or more IPTp doses, in line with WHO recommendations for a minimum of three doses during pregnancy. Non-compliance was defined as receipt of fewer than three doses.

### Data collection and Validation

Data were extracted monthly by two trained personnel using a structured data collection form in EpiCollect5. Extracted data were cross-checked monthly against source registers by the principal investigator. Discrepancies were verified and corrected. Entries recorded as ‘not recorded’ were verified against the original registers before analysis to ensure that the information was absent in the source registers rather than omitted or missed during data extraction.

### Data analysis

Data from EpiCollect5 were exported as csv file and analysed using Stata (StataCorp, College Station, TX, USA). Descriptive statistics were used to summarise the socio-demographic and obstetric characteristics of study participants. Modified Poisson regression with robust variance was used to estimate unadjusted relative risk and adjusted relative risk (aRR) with 95% confidence intervals (CIs) for factors associated with non-compliance with the recommended minimum of three IPTp doses. Variables with more than 50% of observations recorded as ‘not recorded’ were excluded from the multivariable analysis.

### Ethical statement

Ethics approval was obtained from the Sierra Leone Ethics and Scientific Review Committee, Freetown, Sierra Leone (SRC/No: 016/02/2025). Permission to use the data from PCMH was obtained from the Medical Superintendent and the Chief Medical Officer, Department of Obstetrics and Gynaecology at PCMH. Data were anonymised prior to analysis and stored in a password-protected computer accessible only to study investigators.

## RESULTS

A total of 733 pregnant women attended ANC at PCMH during 2024. The mean (standard deviation) age of attendees was 26 (5.3) years, with a range of 14–44 years. Information on marital status, religion, and occupation was missing for more than four fifths of the attendees (Table 1). The mean (standard deviation) weight was 72 kg (15). Among the attendees, 73% (537/733) were primiparous or had one previous birth. Approximately 93% (682/733) of attendees registered their pregnancy after the first trimester. Most women (66%, 483/733) had only one ANC contact, and only 4% (30/733) completed four or more contacts (Table 2).

**TABLE 1.** Socio-demographic characteristics of pregnant women who attended antenatal clinic of Princess Christian Maternity Hospital in Sierra Leone, 2024.

Characteristics	Frequency (%)^A^
Total	733 (100)
Age (in years)	
10–19	60 (8.2)
20–29	487 (66.4)
30–39	174 (23.7)
40–59	9 (1.2)
Not recorded^B^	3 (0.4)
Marital Status	
Married	72 (9.8)
Single/divorced/separated	52 (7.1)
Not recorded^B^	609 (83.1)
Religion	
Christianity	42 (5.7)
Islam	68 (9.3)
Other/no-religion	3 (0.4)
Not recorded^B^	620 (84.6)
Occupation	
Unemployed	37 (5)
Employed	8 (1.1)
Housewife	41 (5.6)
Student	16 (2.2)
Not recorded^B^	631 (86.1)

A Column percentage.

B ‘Not recorded’ indicates numbers not filled in the registers at the time of data entry.

**TABLE 2.** Obstetric characteristics and receipt of intermittent preventive therapy (IPTp) among pregnant women who attended the antenatal clinic of Princess Christian Maternity Hospital in Sierra Leone, 2024.

Characteristics	Frequency, N (%)^A^
Total	733 (100)
Parity^B^	
0	300 (40.9)
1	237 (32.3)
2	117 (16.0)
≥3	76 (10.4)
Gravida^B^	
1	261 (35.6)
2	240 (32.7)
3	129 (17.6)
≥4	100 (13.6)
Total number of ANC visits^B^	
One visit	483 (65.9)
Two to three visits	216 (29.5)
≥4 visits	30 (4.1)
Gestational age at 1st ANC visit (weeks)	
1st trimester (less than 12 weeks)	51 (7)
2nd trimester (13–24 weeks)	358 (48.8)
3rd trimester (25 weeks and above)	324 (44.2)
Receipt of IPTp^C^	
No doses	327 (44.6)
Received 1st dose (IPTp1)	406 (55.4)
Received 2nd dose (IPTp2)^D^	192 (47.3)
Received 3rd dose (IPTp3)^E^	62 (32.3)
Gestational age of IPTp1 (weeks)^D^	
13–24	228 (56.2)
25–36	157 (38.7)
37–39	21 (5.2)

A Column percentages are presented.

B ‘Not recorded’ values were excluded from the denominator; hence, total numbers vary across variables.

C Totals exceed 733.

D Proportion calculated among women who received the first dose of IPTp.

E Proportion calculated among women who received the second dose of IPTp.

### Receipt of doses and compliance to IPTp

Among the 733 attendees, 406 (55%) attendees received the first dose of IPTp (IPTp1). Among the 406 attendees who received the first dose, less than half (47%, 192/406) received the second dose (IPTp2), and of the 192 women who received IPTp2, only 32% (62/192) completed the third dose (IPTp3). Overall, among the 733 attendees, only 62 (8%) women received three or more doses (Table 2). The maximum number of doses received was six (by a single woman).

Among 406 women who received at least one IPTp dose, 18% of those initiating in the second trimester and 12% of those initiating in the third trimester completed three or more doses. Similarly, among 51 women who registered in the first trimester, 13.7% received ≥3 IPTp doses compared with 8.1% among 682 who registered in the second or third trimester (Figure). None of these findings were statistically significant.

**FIGURE.**
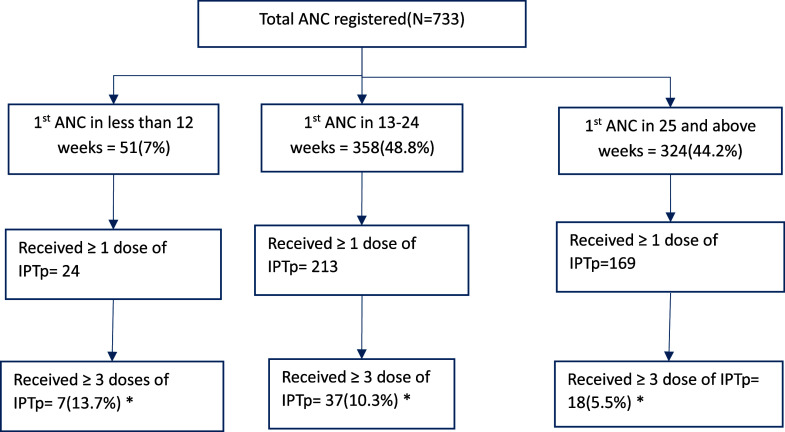
IPTp uptake by gestational age of first antenatal visit among pregnant women who attended the antenatal clinic of Princess Christian Maternity Hospital (PCMH) in Sierra Leone, 2024. *Denominator is the number of pregnant women who had first ANC in the respective trimester.

### Factors associated with non-compliance

In multivariate analysis, only total number of antenatal contacts was found significant. Women with less than four ANC contacts had a 2.8 times higher risk of non-compliance with IPTp3 (aRR: 2.8, 95% CI: 1.5–5.0) compared with those completing four or more contacts (Table 3).

**TABLE 3.** Demographic and obstetric characteristics associated with the non-compliance to recommended minimum three doses of intermittent preventive therapy (IPTp) among pregnant women who attended the antenatal clinic of Princess Christian Maternity Hospital in Sierra Leone, 2024.

Characteristics	Total	Non-compliance to IPTp^A^		RR^B^ (95% CI)	aRR^C^	(95% CI)^D^	
	N	N	n/N (%)^E^			Upper limit–lower limit	
Age group (in years)							
Less than 24	308	282	91.6				
25 and above	425	389	91.5	1 (1–1.1)	1	1	1
Parity							
<3	654	601	91.9				
≥3	79	70	88.6	1 (1–1.1)	1	1	1.1
Gravida							
<4	630	581	92.2				
≥4	100	88	88	1.1 (1–1.1)	1	0.8	1.1
Gestation age at 1st ANC attendance (in weeks)							
1st and 2nd trimesters	409	365	89				
3rd trimester	324	306	94	1 (1–1.1)	1	1	1.1
Total ANC visits							
≥4 visits	30	10	33.3				
<4 visits	699	657	94	**2.8 (2–4.7)**	**2.7**	**1.6**	**4.5**

CI = confidence interval.

A Uptake of doses less than three IPTp was considered non-compliance.

B Unadjusted relative risk.

C Adjusted relative risk.

D Generalised binomial model using modified Poisson with robust variance.

E Row percentages are presented.

Bold values are statistically significant (*P* < 0.05).

## DISCUSSION

We assessed receipt of IPTp doses and compliance with the recommended minimum of three doses at a tertiary care hospital in Freetown, Sierra Leone. This study identified four key findings. First, fewer than 5% of ANC attendees completed the WHO-recommended minimum of four ANC contacts.^1^ Second, only about half of pregnant women received the first dose of IPTp, and receipt declined progressively with subsequent doses. Third, compliance with the recommended three-dose regimen was low, with only one in 12 women completing three or more doses. Finally, women who completed four or more ANC visits showed markedly better IPTp compliance than those with fewer visits.

As a national tertiary referral centre, PCMH receives a large proportion of high-risk pregnancies, late referrals, and women who may have bypassed primary-level ANC services. Many women arrive at the facility in the second or third trimester for specific assessment or complication management, which reduces the number of opportunities to administer IPTp doses. In addition, the high patient volume and complex referral flow of a tertiary maternity hospital may limit the consistency with which IPTp eligibility is reviewed at each visit. These characteristics of a referral-level setting likely contributed to the low proportion of women completing four ANC contacts and the sharp drop in IPTp uptake after the first dose observed in this study. The association between four or more ANC contacts and higher IPTp compliance observed in this study is consistent with evidence from other African countries. Multi-country analyses and malaria indicator survey data have shown that women who initiated ANC early and completed four or more visits are significantly more likely to receive three or more IPTp doses.^9^ Studies from Malawi and Tanzania have also found that limited ANC attendance reduces the likelihood of receiving subsequent doses.^10–12^ Evidence from Ghana similarly demonstrated that IPTp adherence improves with adequate ANC visits and appropriate counselling during consultations.^13^ The 2019 Sierra Leone study also reported low IPTp3 uptake and identified late ANC initiation and fewer ANC visits as key determinants of non-compliance.^14^ Our findings extend this evidence to a tertiary care context, illustrating that low ANC contact frequency continues to limit IPTp delivery even at the highest referral level.

The study has several strengths. It was based on routine antenatal register data collected over a year, reflecting real-world service delivery. Data were extracted by trained personnel and validated monthly by the principal investigator to ensure completeness and consistency. The use of routinely collected data provided a comprehensive picture of IPTp dose receipt in a tertiary care setting. The study was reported in accordance with the Strengthening the Reporting of Observational Studies in Epidemiology (STROBE) guideline.^15^ The study also has several limitations. First, as the data were obtained from a single tertiary hospital, the findings cannot be generalised to other levels of care. Second, as a tertiary referral facility, PCMH receives women who may have already attended ANC at PHUs, but these earlier visits and IPTp doses may not be documented in the referral registers. Thus, women recorded as initiating ANC in later trimesters may be late referrals, and some who did not return for subsequent doses at PCMH may have received them elsewhere. As a result, the receipt of IPTp doses and compliance with recommended three doses observed in this study may underestimate true IPTp coverage. This limitation underscores the need for improved data transfer and follow-up systems to ensure continuity of ANC information across facilities. In addition, a considerable proportion of socio-demographic variables (marital status, religion, and occupation) were not recorded in the registers, although these factors have been identified as influential in previous studies.^9,13,14^ These variables were excluded from the multivariable analysis due to high levels of missing data. Consequently, the analysis primarily reflects service-related factors and may not fully capture individual socio-demographic determinants of non-compliance.

Nevertheless, the study provides important operational insights that can inform programmatic efforts to improve compliance with the recommended minimum of three IPTp doses. First, the very low completion of four ANC contacts suggests that strengthening follow-up mechanisms may support improved continuity of care. Second, the decline across IPTp doses suggests potential missed opportunities for IPTp delivery during ANC contacts. The findings highlight the importance of consistent counselling on the need for repeat visits, particularly for women referred from peripheral facilities. Finally, this study illustrates how routine facility data can be used to identify implementation gaps and inform local programmatic decision-making.

Future work should examine referral documentation systems and how ANC information is transferred from PHUs to tertiary hospitals. Training ANC providers to consistently review IPTp eligibility at each contact and on complete documentation is also needed. In addition, further studies exploring the reasons for reduced receipt and the decline in IPTp dose completion after the first dose would provide insights into strengthening IPTp delivery at tertiary-level facilities.

## CONCLUSION

Receipt of IPTp doses and compliance with the recommended minimum of three doses were low in this tertiary hospital setting and were associated with fewer ANC contacts. These findings highlight gaps in routine service delivery and suggest that improving continuity of ANC contacts may support better compliance with IPTp recommendations. Further evaluation in broader settings is warranted.
